# Reversing the Warburg Effect: YW3-56 Induces Leukemia Differentiation via AKT-Mediated Glucose Metabolic Reprogramming

**DOI:** 10.3390/ph18111646

**Published:** 2025-10-31

**Authors:** Di Zhu, Dan Gao, Yu Lu, Na Chen, Li Zhang, Lan Zhang, Yuji Wang

**Affiliations:** 1 Department of Pharmacy, Xuanwu Hospital, Capital Medical University, Beijing 100053, China; zhudi950928@163.com (D.Z.); gaodan_gd@163.com (D.G.); chennaaa@126.com (N.C.); 13651194287@163.com (L.Z.); 2 Department of Medicinal Chemistry, College of Pharmaceutical Sciences, Capital Medical University, Beijing 100069, China; luyu@mail.ccmu.edu.cn; 3 Beijing Key Laboratory of Drug Innovation for Neuro-Oncology, Beijing Engineering Research Center of Targeted Drugs and Cell Therapy for CNS Tumors, Beijing 100069, China

**Keywords:** PAD4 inhibitor, differentiation, glucose metabolism, transcriptomics, proteomics, mass cytometry

## Abstract

**Background**: Protein arginine deiminase 4 (PAD4) has emerged as a promising therapeutic target for acute promyelocytic leukemia (APL) because of its role in epigenetic regulation and leukemogenesis. All-trans retinoic acid, a standard differentiation agent in APL therapy, has been shown to upregulate PAD4 expression during leukemic cell maturation. Interestingly, first-generation PAD4 inhibitors also promote differentiation, but simultaneously trigger compensatory PAD4 overexpression, underscoring the unresolved complexity of PAD4 modulation in leukemia therapy. **Methods**: In this study, we employed mass cytometry and transcriptomic–proteomic integrated analysis to investigate the underlying mechanisms of YW3-56, a dual-function PAD4 inhibitor against protein expression and enzymatic function, in NB4 leukemia cells. Functional validation was conducted using Western blot and metabolic assays. **Results**: Mass cytometry analysis revealed that YW3-56 reduced leukemia stemness (CD44/CD133), while enhancing myeloid differentiation (CD11b/CD14) and immunogenic activation (CD80/CD86). Multiomics analysis revealed a YW3-56-induced metabolic shift characterized by downregulation of glycolytic enzymes and upregulation of the tricarboxylic acid cycle and pentose phosphate pathway components, indicating a reversal of the Warburg effect. Mechanistically, this metabolic reprogramming was driven by reduced AKT expression and phosphorylation at Thr308, impaired GLUT1 expression and membrane localization, and decreased glucose uptake, which collectively promoted the differentiation of NB4 cells. Additionally, YW3-56 suppressed the downstream mTOR pathway, inducing caspase-3/PARP-mediated apoptosis and inhibiting cell proliferation. **Conclusions**: Our study demonstrated that YW3-56 exerts multimodal antileukemic effects in APL by simultaneously targeting PAD4-mediated epigenetic regulation, AKT-driven metabolic reprogramming and cellular differentiation, highlighting PAD4-AKT signaling as a promising target for APL combination therapy.

## 1. Introduction

Acute promyelocytic leukemia (APL), a distinct and aggressive subtype of acute myeloid leukemia, is characterized by differentiation arrest at the promyelocytic stage, leading to pathological accumulation of immature promyelocytes [1,2]. While current differentiation therapies—mainly all-trans retinoic acid (ATRA) and arsenic trioxide (ATO)—have revolutionized APL management, they may have serious side effects, including differentiation syndrome and hyperinflammation, thus leading to a poor prognosis and a high risk of recurrence, particularly in elderly patients [3,4,5]. These unresolved challenges underscore the urgent need to develop novel therapeutic strategies and deepen our understanding of the molecular mechanisms governing APL cell differentiation.

Emerging evidence highlights peptidyl arginine deiminase 4 (PAD4) as a potential therapeutic target in myeloid malignancies. This epigenetic modifier catalyzes the citrullination of arginine residues in histones and other nuclear proteins [6,7], playing multifaceted roles in neutrophil extracellular trap (NET) formation, tumor progression, and immune evasion [8,9,10,11]. Intriguingly, previous studies have shown that ATRA induces the differentiation of APL cells and increases PAD4 expression [12,13]. Notably, first-generation PAD4 inhibitors (e.g., F-/Cl-amidine) also induce leukemia cell differentiation and trigger compensatory PAD4 overexpression [14]. Given the complex regulatory networks involving PAD4 in myeloid differentiation, further exploration of the mechanisms through which PAD4 inhibitors induce leukemia cell differentiation is highly important.

Metabolic reprogramming is an important feature in the initiation and progression of acute myeloid leukemia and plays a crucial role in regulating the differentiation and activation of both cancer cells and leukemia stem cells (LSCs) [15,16,17]. Notably, APL cells exhibit a marked dependency on aerobic glycolysis (“Warburg effect”) to sustain their undifferentiated state and survival [18,19], with LSCs demonstrating increased glucose uptake compared with their differentiated counterparts [20]. Growing evidence has underscored the strong link between metabolic reprogramming and cancer stemness [21,22,23]; however, the potential crosstalk between PAD4-mediated epigenetic regulation and metabolic pathways in APL differentiation remains unexplored.

This study aimed to investigate the metabolic alterations that occur during PAD4 inhibitor (YW3-56)-induced differentiation of NB4 acute promyelocytic leukemia cells, and to examine their potential associations with key signaling pathways such as AKT/mTOR. By employing an integrative multiomics approach encompassing mass cytometry, transcriptomics, and proteomics analysis, we systematically characterized the differentiation-associated metabolic shifts in PAD4 inhibitor-treated APL cells at single-cell resolution and reveal key regulatory nodes connecting citrullination signaling with metabolic pathways. Our findings may suggest new therapeutic targets and strategies for acute promyelocytic leukemia.

## 2. Results

### 2.1. The PAD4 Inhibitor YW3-56 Suppresses Cell Viability in APL Cells

**Figure 1:**
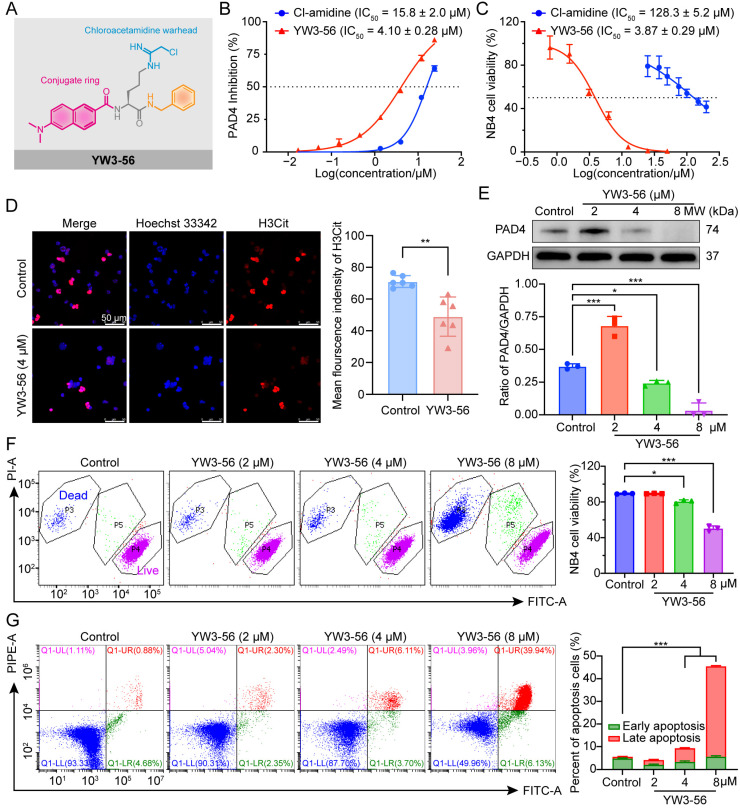
The PAD4 inhibitor YW3-56 inhibits NB4 cell proliferation and induces apoptosis by inhibiting H3Cit modification. (**A**) Chemical structure of YW3-56. Chloroacetamidine warhead: key moiety that covalently binds to the catalytic residues in the PAD4 active site, leading to irreversible inhibition; Conjugate ring: enhancing binding affinity and selectivity toward the PAD4 protein. (**B**) PAD4 enzymatic activity measured by a colorimetric assay. (**C**) Effect of YW3-56 on NB4 cell viability measured by 3-(4,5-Dimethylthiazol-2-yl)-2,5-Diphenyltetrazolium Bromide (MTT) assay. (**D**) Immunofluorescence confocal microscopy analysis of H3Cit expression (red) in NB4 cells treated with 4 μM YW3-56, with statistical evaluation performed using Student’s two-tailed *t*-test. Nuclear are stained with Hoechst 33342 (blue). Scale bar: 50 μm. (**E**) Western blot analysis of PAD4 expression in NB4 cells treated with YW3-56 (8, 4, and 2 μM). GAPDH was used as a loading control. (**F**) Cell viability assessed via flow cytometry by Calcein-AM/PI staining. (**G**) Apoptosis analysis using Annexin V-FITC/PI staining, showing a dose-dependent increase in both early apoptotic (Annexin V+/PI−) and late apoptotic (Annexin V+/PI+) cell populations. Unless otherwise specified, data were analyzed using one-way ANOVA Tukey’s test and presented as means ± SD. *, *p* < 0.05; **, *p* < 0.01; ***, *p* < 0.001. one-way ANOVA Tukey’s test or Student’s two-tailed *t*-test.

YW3-56 (chemical structure and characteristics shown in Figure 1A and Figure S1) is a potent PAD4 inhibitor with an half maximal inhibitory concentration (IC_50_) of 4.10 ± 0.28 μM, demonstrating 3.9-fold greater in vitro inhibitory activity—as measured by Histone 3 citrullination (H3Cit) levels—compared to the first-generation inhibitor Cl-amidine (Figure 1B and Figure S2A). Notably, YW3-56 exhibited stronger anti-proliferative effects in human promyelocytic leukemia NB4 cells, with an IC_50_ of 3.87 ± 0.29 μM, which is 33.2-fold lower than that of Cl-amidine (Figure 1C and Figure S2B). Immunofluorescence analysis revealed that treatment with 4 μM YW3-56 effectively suppressed H3Cit expression in NB4 cells (Figure 1D). Importantly, unlike first-generation PAD4 inhibitors that induce feedback upregulation of PAD4 in APL cells, Western blot analysis confirmed that YW3-56 reduced PAD4 protein level in a dose-dependent manner, with the most pronounced suppression observed at higher concentrations, consistent with our previous findings (Figure 1E) [24].

To further evaluate the cytotoxic effects of YW3-56 on NB4 cells, we performed Calcein-AM/PI dual-staining assays, which revealed concentration-dependent reductions in the proportion of viable NB4 cells (Figure 1F). Intriguingly, increasing YW3-56 concentrations induced a progressive shift from viable (Calcein-AM+, green fluorescence) to late apoptotic (PI+, red fluorescence) populations, with an intermediate transitional cell population observed. These findings suggest that YW3-56 inhibits NB4 cell proliferation and induces cell death, potentially through downregulation of PAD4 expression and subsequent suppression of H3Cit modification.

### 2.2. YW3-56 Induces Dose-Dependent Apoptosis in APL Cells

To further determine the cell death modality induced by YW3-56, we performed Annexin V-FITC/PI dual-staining assays (Figure 1G). Treatment with YW3-56 (2, 4, and 8 μM) for 24 h resulted in a dose-dependent increase in the proportion of apoptotic cells, with the proportion of early apoptotic cells increasing from 2.14 ± 0.22% (2 μM) to 5.73 ± 0.35% (8 μM), whereas the proportion of late apoptotic cells exhibited a more pronounced increase from 2.11 ± 0.20% (2 μM) to 39.84 ± 0.19% (8 μM), suggesting progressive apoptosis induction at higher concentrations. Consistent with these findings, mass cytometry analysis (Figure S3) revealed that 8 μM YW3-56 significantly upregulated cleaved caspase-3 (5.5-fold) and cleaved PARP (3.8-fold), confirming the activation of the intrinsic apoptotic pathway. These data collectively establish that YW3-56 triggers caspase-dependent apoptosis, predominantly late-stage apoptosis, in NB4 cells.

To extend these observations, we evaluated the effects of YW3-56 in another APL model, the HL-60 cell line. Consistent with its activity in NB4 cells, YW3-56 demonstrated strong growth-inhibitory effects in HL-60 cells, yielding an IC_50_ of 2.27 ± 0.17 μM (Figure S4A). Apoptosis analysis further revealed a clear dose-dependent response, with pronounced late-stage apoptosis observed at higher concentrations (4 μM and 8 μM; Figure S4B,C). Collectively, these results suggest that YW3-56 has therapeutic potential for APL by inhibiting proliferation and inducing late-stage apoptosis.

### 2.3. Mechanistic Insights into PAD4 Inhibitor-Induced Differentiation and Immune Activation

**Figure 2:**
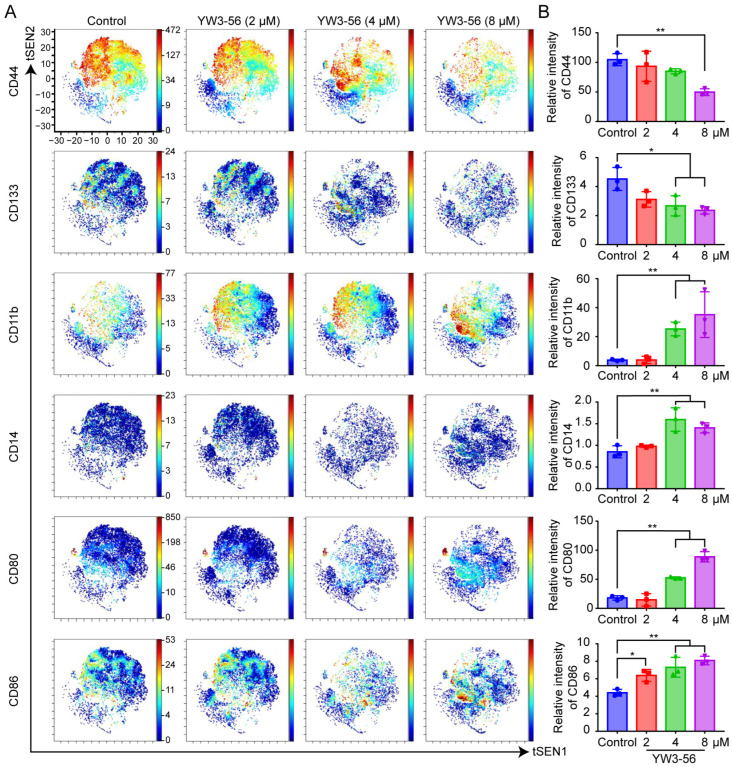
YW3-56 inhibited stemness properties but induced NB4 cell differentiation and immune activation. (**A**) t-distributed stochastic neighbor embedding (tSNE) plot showing associated protein expression. (**B**) Statistical analysis of the expression of stemness-, differentiation- and immune-related proteins. Mean signal intensity was normalized to a bead standard for protein quantification. The data are shown as the means ± SD. *, *p* < 0.05; **, *p* < 0.01.

To systematically characterize the phenotypic remodeling induced by PAD4 inhibition, we performed mass cytometry analysis on NB4 cells via a 33-marker panel (Table S1). The marker selection was guided by previously reported pathways associated with PAD4 inhibition and the established differentiation features of APL as described in the literature [6,25,26]. As shown in Figure 2A,B, YW3-56 treatment significantly suppressed the expression of stemness-associated proteins, including CD44 and CD133, in a concentration-dependent manner. These markers are commonly upregulated in cancer stem cells across various malignancies [27,28]. Treatment with 4 μM and 8 μM YW3-56 led to a marked increase in the expression of the differentiation markers CD11b and CD14 in NB4 cells. These results suggest that the PAD4 inhibitor YW3-56 can attenuate stemness-related behaviors and promote the differentiation of leukemic cells. Notably, while first-generation inhibitors such as Cl-amidine induce compensatory PAD4 overexpression during differentiation [14], our data suggest that the critical determinant for NB4 cell differentiation is the inhibition of H3Cit modification (PAD4 enzymatic activity) rather than the inhibition of PAD4 protein expression. These findings establish a nonlinear relationship between PAD4 expression and differentiation efficiency.

In addition, YW3-56 treatment induced a gradual transition of NB4 cells from an undifferentiated state to a distinct CD80^+^ and CD86^+^ state, which is indicative of enhanced immune activation, particularly T-cell activation [29,30]. These findings demonstrate that YW3-56 increases immunogenic potential while driving leukemic cell differentiation, positioning YW3-56 as a dual-function agent that targets both stemness ablation and immune microenvironment modulation. However, the precise molecular mechanism mediating this dual regulatory axis remains to be fully elucidated.

To further evaluate the differentiation-inducing potential of YW3-56, we compared its effects with the standard agent ATRA and explored their combined therapeutic potential. In vitro viability assays (Figure S5A) showed that ATRA at 10 μM modestly inhibited NB4 cell proliferation, while co-treatment with YW3-56 maintained the cytotoxic activity of YW3-56. Flow cytometry analysis (Figure S5B,C) revealed that 4 μM YW3-56 markedly increased both CD11b^+^ and CD14^+^ NB4 cells after five days, consistent with mass cytometry findings and indicating its capacity to induce bidirectional differentiation 170 into granulocytic- and monocytic-like lineages. In contrast, 10 nM ATRA significantly increased CD11b expression without affecting CD14, confirming its granulocytic differentiation effect, as reported previously [31,32]. Notably, the combination of YW3-56 and ATRA reduced CD11b^+^ cells while strongly enhanced CD14^+^ cells, suggesting a shift from ATRA-driven granulocytic toward monocytic differentiation. Collectively, these findings 175 highlight the potential of YW3-56 in combination with ATRA as a novel therapeutic approach for APL, offering effective suppression of leukemic cell growth while redirecting differentiation to the monocytic lineage, which may help mitigate ATRA-associated differentiation syndrome and reduce early mortality.

### 2.4. Quality Control and Correlation Analysis of Transcriptomes and Proteomes

To investigate the potential mechanism of NB4 cell differentiation following PAD4 inhibition, transcriptomic and proteomic profiling of cells treated with YW3-56 (4 μM, a concentration that induces pronounced phenotypic effects with limited apoptotic interference) or PBS (control) was performed. As shown in Table 1, a total of six samples (two conditions with three biological replicates) were subjected to RNA sequencing, yielding approximately 272 million raw reads. After filtering the reads on the basis of quality, more than 97% of the RNA sequencing reads were retained for downstream analysis. High-quality sequencing data were available for all the samples, with average Q20 and Q30 scores of 97.35% and 92.50%, respectively. In addition, iTRAQ proteomics identified a total of 25,437 peptides, 12,888 proteins and 4074 protein groups (FDR < 0.01). These results confirm the reliability and usability of the dataset.

**Figure 3:**
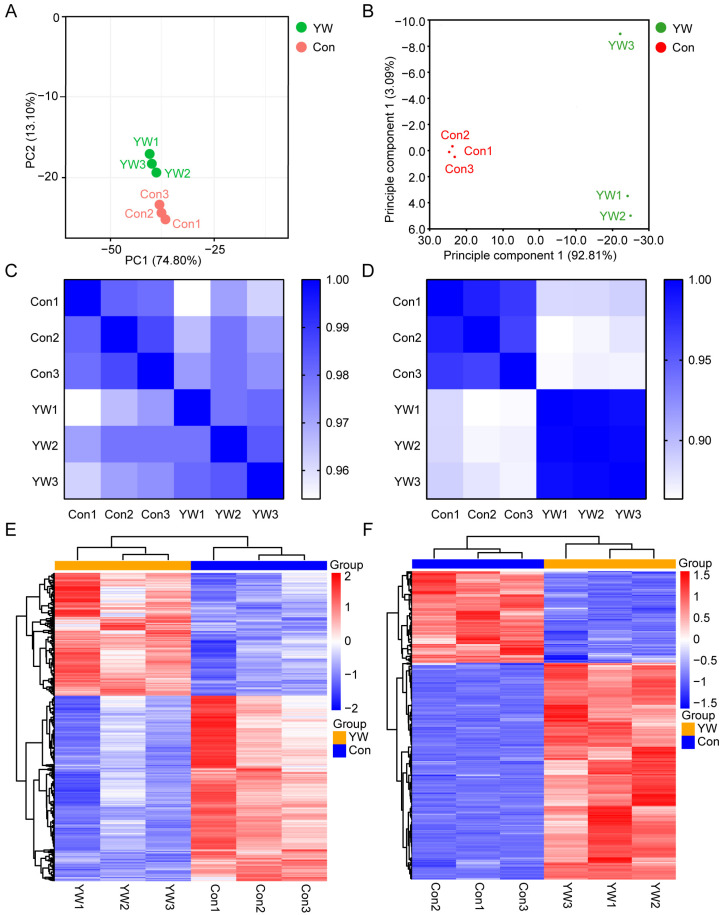
Transcriptome and proteome analysis of YW3-56-treated NB4 cells (Con, control; YW, YW3-56). (**A**) PCA plots, (**C**) Pearson’s correlation analysis, and (**E**) Heatmap of differentially expressed mRNAs from the transcriptomic data. (**B**) PCA plots, (**D**) Pearson’s correlation analysis, and (**F**) Heatmap of differentially expressed proteins from the proteomic data.

Principal component analysis (PCA) of the transcriptomic and proteomic datasets revealed 87.9% and 95.9% cumulative variance in the first two principal components, respectively, indicating clear spatial segregation between the YW3-56-treated and control groups (Figure 3A,B). High intragroup reproducibility was confirmed by Pearson correlation analysis (Figure 3C,D). Hierarchical clustering analysis revealed clear differences in the transcriptome and proteome in response to PAD4 inhibition (Figure 3E,F), with minimal variability observed among the biological replicates. These results indicate that YW3-56 treatment induces significant and stable alterations in both gene and protein expression levels in NB4 cells.

### 2.5. Integrated Multiomics Analysis Revealed That YW3-56 Reprograms Glucose Metabolism

**Figure 4:**
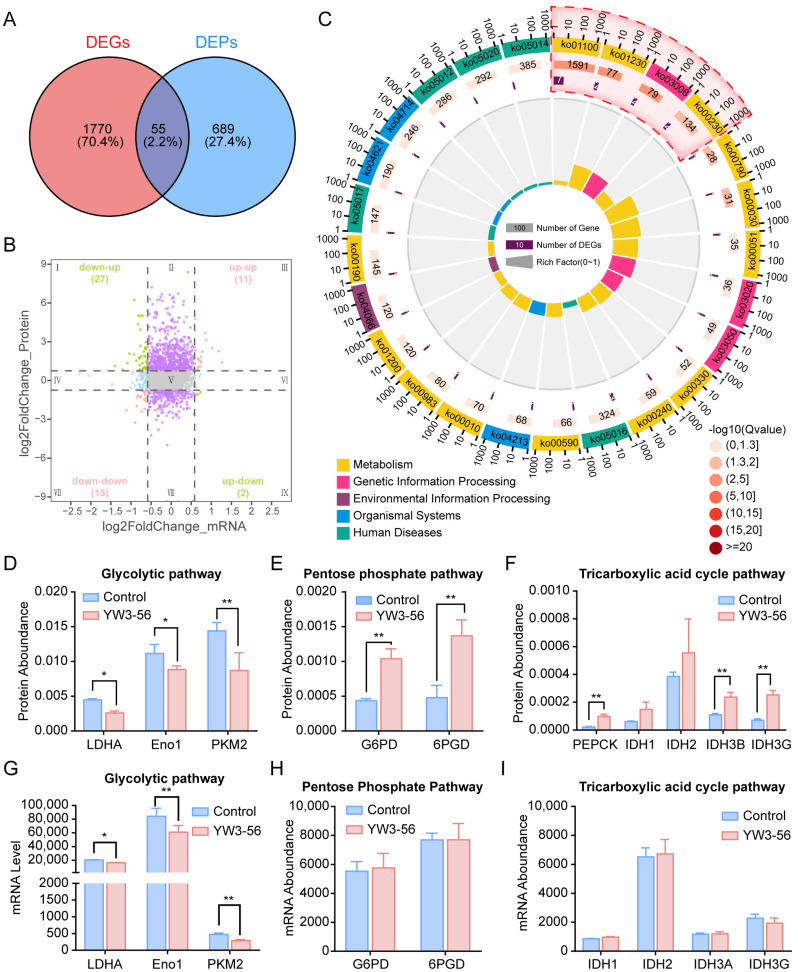
Multiomics profiling revealed YW3-56-mediated metabolic reprogramming. (**A**) Venn diagram showing the overlap among total DEGs/DEPs in YW3-56-treated NB4 cells. (**B**) Nine-quadrant plot illustrating the correlation between transcriptomic and proteomic regulation. Quadrants I and IX: mRNA and corresponding protein levels change in opposite directions, suggesting post-transcriptional or translational regulation (e.g., miRNA-mediated regulation). Quadrants II and VIII: Proteins show differential expression without corresponding mRNA changes. Quadrants III (upregulated) and VII (downregulated): mRNA and protein exhibit concordant differential expression, reflecting coordinated transcriptional and translational regulation. Quadrants IV and VI: mRNA shows differential expression without corresponding protein changes. Quadrants V: Both mRNA and protein are not differentially expressed in most genes. (**C**) Circos plot summarizing the features of coexpressed DEGs/DEPs enriched in the top 25 KEGG pathways. From inner to outer layers: the RichFactor, the number of DEGs, total gene count (with the gradual red scale indicating −log10[q-value] from the enrichment analysis), and the KEGG accession numbers (with different colors representing distinct KEGG classes). Proteomic analyses of key enzymes involved in the (**D**) glycolytic pathway, (**E**) pentose phosphate pathway, and (**F**) tricarboxylic acid cycle. Transcriptomic analyses of key enzymes related to the (**G**) glycolytic pathway, (**H**) pentose phosphate pathway and (**I**) tricarboxylic acid cycle. The data are shown as the means ± SD. *, *p* < 0.05; **, *p* < 0.01.

To better elucidate the regulatory mechanism of YW3-56-induced differentiation, we performed integrated analysis of the transcriptomic and proteomic profiles of YW3-56-treated and untreated NB4 cells and identified 1825 differentially expressed genes (DEGs, |log_2_FC| > 0.6, *p* < 0.05) and 744 differentially expressed proteins (DEPs, |log_2_FC| > 1, *p* < 0.05). Venn diagram analysis revealed that 55 factors were significantly altered at both the RNA level and the protein level (Figure 4A). Nine-quadrant coordinate analysis classified all differential genes/proteins into distinct clusters on the basis of expression dynamics (Figure 4B). Among the 55 factors, 42 were upregulated and 13 were downregulated at the gene level; 17 exhibited decreased enrichment and 38 exhibited increased enrichment at the protein level. Notably, 26 factors exhibited concordant expression trends. The 11 coupregulated factors included PTPRC (a positive regulator of T-cell activation), IFI16 (a hematopoietic differentiation modulator), and SMARCE1 (a chromatin remodeler that enhances tumor suppressor accessibility). The 15 factors co-downregulated factors included ALDOC (a glycolytic enzyme broadly expressed in vertebrates), ARHGAP45, ATP5MF, and CLPB, which are functionally associated with glucose metabolism and ATP synthesis. These findings on coexpressed DEGs/DEPs support the dual regulatory function of YW3-56 and suggest that its effects may involve the reprogramming of glucose metabolism, particularly the suppression of glycolysis.

Gene Ontology (GO) enrichment analysis of the coexpressed DEGs/DEPs revealed the top 25 significantly enriched functional categories, which were visualized in a GO enrichment histogram (Figure S6) and a bubble diagram (Figure S7). The analysis revealed predominant involvement in broad biological processes (e.g., metabolic processes and biological regulation), and molecular functions (e.g., protein binding). These findings suggest that YW3-56 may promote NB4 cell differentiation by modulating both metabolic pathways and epigenetic processes. While its epigenetic effect is consistent with its PAD4 inhibition mechanism, its impact on metabolic pathways offers novel insight into its differentiation-inducing mechanism.

Subsequently, integrated KEGG pathway analysis of coexpressed DEGs/DEPs from YW3-56-treated NB4 cells revealed distinct metabolic reprogramming signatures. Circos plot (Figure 4C) and bubble diagram (Figure S8) analyses demonstrated that co-downregulated factors were enriched in metabolic pathways, genetic information processing, and organismal systems. Specifically, the Circos plot indicated that the Metabolic pathways (ko01100) contained 7 co-downregulated DEGs, while the pathway for Purine metabolism (ko00230) contained 2 concordantly downregulated factors with a high enrichment score, suggesting the existence of an interrelated metabolic regulatory network. Strikingly, prominent enrichment of factors that were downregulated at the gene level but upregulated at the protein level was observed in glucose metabolism modules, such as the tricarboxylic acid (TCA) cycle, 2-oxocarboxylic acid metabolism, and pyruvate metabolism. The differential enrichment patterns further support the role of YW3-56 in metabolic reprogramming and suggest that it may enhance glucose oxidation through activation of the TCA cycle.

To evaluate the specific impact of YW3-56 on glucose metabolism, we analyzed the expression of key enzymes involved in three major glucose metabolism pathways. Proteomic data revealed a notable decrease in the expression of enzymes within the glycolytic pathway, including LDHA, Eno1, and PKM2 (Figure 4D), whereas enzymes in the pentose phosphate pathway, such as G6PD and 6PGD (Figure 4E), and in the TCA cycle, including PEPCK, IDH3B, and IDH3G (Figure 4F), were upregulated. Additionally, gene expression analysis corroborated these results, revealing a reduction in the mRNA levels of glycolytic enzymes (Figure 4G), whereas the mRNA levels of enzymes in the pentose phosphate pathway and the TCA cycle remained unchanged in the intervention group vs. the control group (Figure 4H,I), suggesting that YW3-56 may regulate oxidative metabolic pathways at the posttranscriptional level. Collectively, these findings suggest that YW3-56 may reprogram glucose metabolism by suppressing glycolytic flux and promoting other aerobic metabolic pathways, thereby reversing the Warburg effect in differentiated NB4 cells.

### 2.6. YW3-56 Orchestrates Differentiation and Glucose Metabolism Through PI3K-AKT-mTOR Axis Modulation

**Figure 5:**
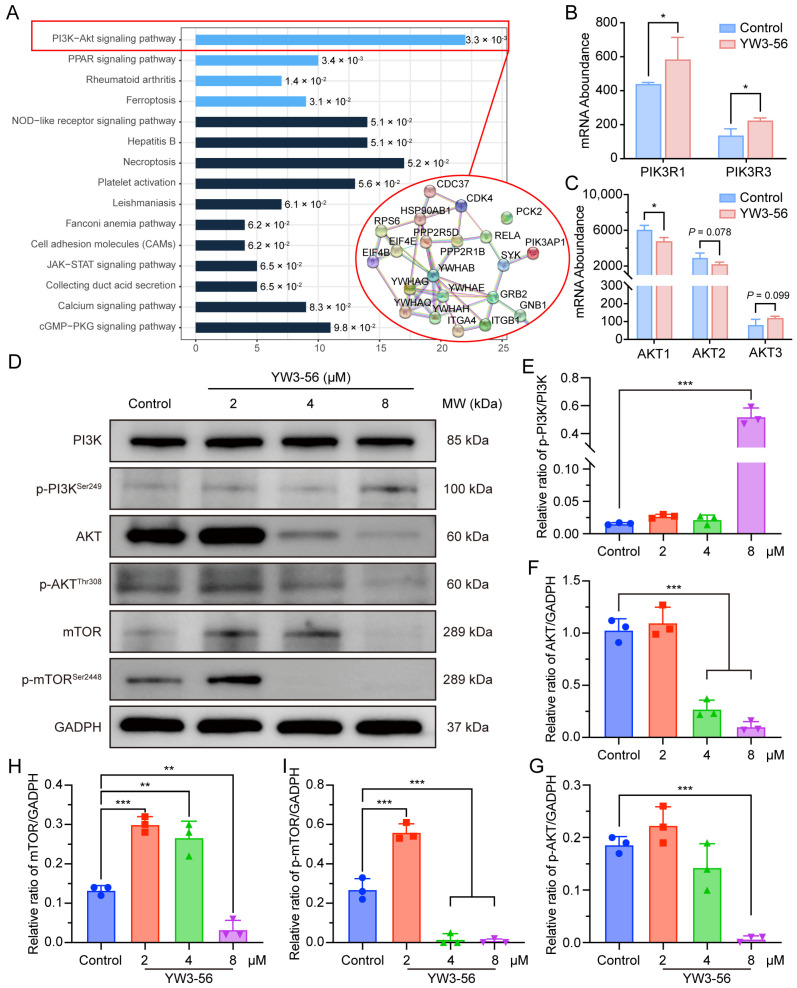
YW3-56 promoted metabolic-differentiation coupling in NB4 cells via PI3K-AKT-mTOR signaling. (**A**) KEGG pathway enrichment analysis of proteomic data highlighting PI3K-AKT signaling modulation. (**B**,**C**) Transcriptomic analysis confirmed the increase in the expression of PI3K/AKT regulatory subunits. (**D**) Representative Western blot images of the PI3K-AKT-mTOR pathway in NB4 cells treated with YW3-56 (8, 4, and 2 μM). GAPDH was used as a loading control. (**E**–**I**) Quantification of the protein expression levels and phosphorylation status of PI3K (Ser249), AKT (Thr308), and mTOR (Ser2448). The data are shown as the means ± SD. *, *p* < 0.05; **, *p* < 0.01; ***, *p* < 0.001.

To further elucidate the molecular mechanism underlying YW3-56-induced differentiation and glucose metabolism regulation in NB4 leukemia cells, we conducted a KEGG pathway enrichment analysis of the coexpressed DEGs/DEPs. The results revealed that YW3-56 significantly affects the enrichment of factors in the PI3K-AKT signaling pathway (Figure 5A), which is a critical pathway regulating diverse cellular processes, including proliferation, survival, and differentiation [33,34,35,36]. Interestingly, multiomics profiling revealed coordinated upregulation of PI3K pathway components, including the PIK3R1/PIK3R3 mRNAs (Figure 5B) and PIK3AP1 protein (Figure S9A), suggesting PI3K activation. However, RNA-seq analysis showed a reduction in AKT mRNA levels, predominantly AKT1 (Figure 5C), while mass cytometry demonstrated that AKT phosphorylation was markedly decreased in NB4 cells treated with 4 μM or 8 μM YW3-56 (Figure S9B). Together, these findings suggest that PAD4 inhibition may exert complex regulatory effects within the PI3K-AKT signaling axis.

To validate these findings, we conducted Western blot analysis to assess the expression of key proteins in the PI3K/AKT signaling cascade (Figure 5D). Quantification revealed a significant increase in p-PI3K (Ser249) levels in NB4 cells treated with 8 μM YW3-56 (Figure 5E), corroborating the multiomics results and confirming the activation of PI3K. Further analysis revealed that YW3-56 dose-dependently decreased the levels of total AKT and phosphorylated AKT (Thr308) while inhibiting mTOR/p-mTOR (Ser2448) at the 8 μM dose (Figure 5F–I). These results suggest that YW3-56 may regulate glucose metabolism and induce differentiation in NB4 cells by selectively suppressing the expression and activation of AKT and mTOR, while concurrently triggering PI3K compensatory activation, possibly through the release of IRS-mediated signaling normally suppressed by mTORC1/S6K [37,38].

### 2.7. YW3-56 Disrupts Glucose Uptake via GLUT1 Downregulation

**Figure 6:**
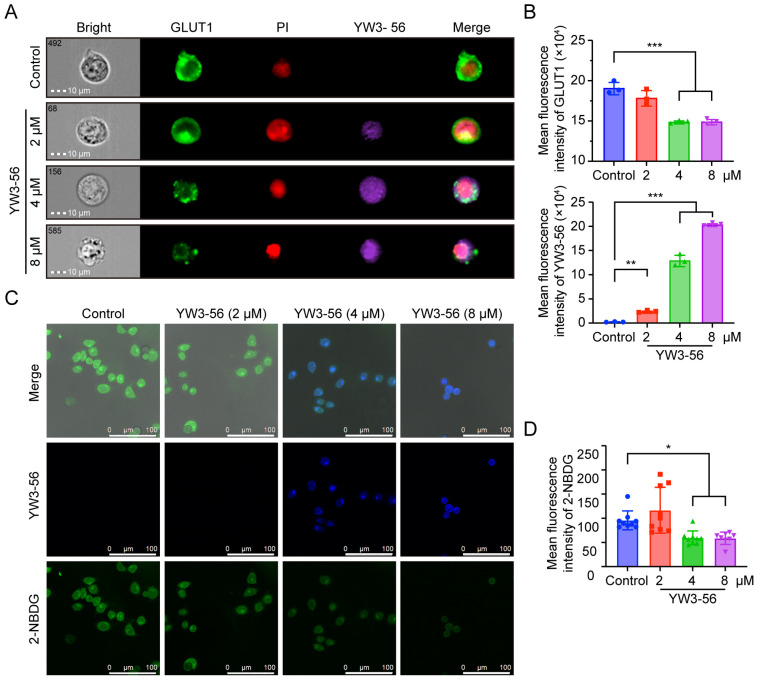
Inhibition of glucose uptake in NB4 cells via GLUT1 by YW3-56. (**A**,**B**) Representative immunofluorescence images and statistical analysis from imaging flow cytometry showing GLUT1 (green) expression and membrane localization in NB4 cells treated with YW3-56 (purple). Nuclei were counterstained with PI (red). (**C**,**D**) Confocal microscopy images and statistical analysis of 2-NBDG (green) glucose uptake in NB4 cells treated with YW3-56 (blue). The data are shown as the means ± SD. *, *p* < 0.05; **, *p* < 0.01; ***, *p* < 0.001.

Compared with non-LSCs, LSCs exhibit markedly elevated glucose uptake mediated by glucose transporter 1 (GLUT1), thereby sustaining the hypermetabolic state of APL cells [39,40,41]. To verify whether YW3-56 induces NB4 cell differentiation by altering glucose metabolism, we employed high-content imaging flow cytometry to quantify GLUT1 expression and subcellular localization. As shown in Figure 6A,B, the green GLUT1 signal in NB4 cells significantly decreased upon treatment with 4 μM and 8 μM YW3-56, with a corresponding reduction in membrane localization. These findings suggest that YW3-56 exerts a suppressive effect on GLUT1 expression and may impede its translocation to the cell membrane.

Consistent with GLUT1 downregulation, we detected the suppression of 2-NBDG glucose uptake (Figure 6C,D), suggesting that YW3-56 impairs glucose uptake in NB4 cells, which may lead to metabolic stress and apoptosis induction. Given that PAD4 inhibition leads to the suppression of both AKT expression and activation, AKT inactivation may disrupt critical downstream processes such as the translocation of GLUT1 to the plasma membrane [42,43]. These findings suggest a potential PAD4-H3Cit-AKT-GLUT1 axis in APL cells, linking epigenetic regulation to metabolic reprogramming and leukemic cell differentiation.

## 3. Discussion

Early studies established that ATRA-induced differentiation of acute promyelocytic leukemia cells coincides with upregulated PAD4 protein expression [12,13]. Concurrently, first-generation PAD4 inhibitors (F-/Cl-amidine), although capable of inducing leukemic differentiation, trigger compensatory overexpression of PAD4 [14]. These findings obscure the precise role of PAD4 in differentiation-inducing therapies and suggest a complex regulatory mechanism underlying the function of PAD4 in leukemogenesis.

In this study, we provided mechanistic insights into the multimodal antileukemic effects of YW3-56, a novel PAD4 inactivator with dual inhibitory activity against both protein expression and enzymatic function. Unlike first-generation PAD4 inhibitors inducing differentiation at the cost of compensatory PAD4 overexpression, YW3-56 effectively suppresses H3Cit modification, a PAD4-dependent epigenetic modification, without triggering negative feedback. Moreover, YW3-56 induces a dose-dependent transition from viable to apoptotic states in NB4 cells, with a marked increase in late apoptosis. Mass cytometry further revealed that YW3-56 treatment simultaneously suppresses leukemia stemness (CD44/CD133 downregulation) and promotes myeloid differentiation (CD11b/CD14 upregulation), as well as enhances immunogenic activation (CD80/CD86 increase). Notably, our findings establish that PAD4 inhibition, rather than PAD4 protein abundance modulation, functions as a critical epigenetic regulator of leukemia cell differentiation. This effect may act in part through the modulation of PAD4 downstream p53 target genes, whereby p53 orchestrates both apoptotic signaling (e.g., Bax, PUMA) and metabolic regulation (e.g., TIGAR, SCO2) to jointly shape cell fate and energy homeostasis [44,45,46].

A subsequent multiomics analysis identified 55 factors consistently altered at both RNA and protein levels in YW3-56-treated NB4 cells. Co-upregulated factors, such as PTPRC and IFI16, were associated with immune activation and differentiation, whereas co-downregulated factors, including ALDOC, ARHGAP45, ATP5MF, and CLPB, were primarily linked to energy metabolism. These co-expressed DEGs/DEPs highlight the dual regulatory effects of YW3-56 and point to metabolic reprogramming—particularly glycolytic suppression—as a potential mechanism. Building on this, multiomics pathway analysis further showed that YW3-56 could reprogram glucose metabolism, characterized by the suppression of glycolytic enzymes (e.g., LDHA, Eno1, and PKM2) alongside the upregulation of pentose phosphate pathway components (e.g., G6PD and 6PGD) and TCA cycle components (e.g., PEPCK, IDH3B, and IDH3G), indicative of a reversal of the Warburg effect. Mechanistically, this metabolic shift may be correlated with the inhibition of AKT, a key regulator of glycolysis [43,47,48]. YW3-56 treatment significantly decreased AKT1 mRNA levels and substantially reduced both total AKT expression and phosphorylation at Thr308, thereby disrupting the expression and plasma membrane translocation of the downstream glucose transporter GLUT1. This impairment reduced glucose uptake, reprogrammed cellular energy metabolism, and promoted the differentiation of leukemia cells into more mature and specialized phenotypes. Additionally, YW3-56 inhibited the downstream mTOR signaling cascade, resulting in the activation of caspase-3/PARP-dependent apoptosis and a pronounced suppression of leukemia cell proliferation. Despite the suppression of AKT/mTOR signaling, the level of phosphorylated PI3K was increased, most likely reflecting a well-established negative-feedback loop in which mTORC1/S6K normally suppresses IRS-mediated PI3K activation; inhibition of mTORC1/S6K releases this restraint and induces compensatory PI3K activation [37,38]. Notably, previous studies have shown that pharmacological inhibition of PAD enzymes, including PAD4, decreases AKT phosphorylation [49], whereas PAD4 upregulation can activate the PI3K/AKT pathway [26]. In addition, PAD4 has been reported to functionally interact with p53, a key regulator of PI3K/AKT signaling [50,51]. Taken together with our findings, these observations suggest the potential existence of a PAD4-H3Cit-AKT-GLUT1 axis that links epigenetic regulation to metabolic reprogramming and leukemic differentiation.

Nevertheless, several important limitations need to be considered. First, most functional validations were performed in NB4 cells, and thus the generalizability of YW3-56’s effects across other APL subtypes remains to be determined. Second, although our integrated single-cell and multiomics analyses, together with functional validation, indicate a potential link between cellular phenotypes and molecular pathways, this remains inferential. To substantiate these associations, future studies will isolate distinct subpopulations for targeted omics profiling and pseudotime analysis. Finally, in vivo validation in relevant APL murine models or primary APL patient samples is essential to evaluate the therapeutic efficacy, toxicity, and pharmacokinetics of YW3-56 and combinatorial potential with established differentiation-inducing agents (e.g., ATRA and ATO), which will be essential for determining its clinical relevance and feasibility of synergistic treatment strategies.

In conclusion, our integrated analysis demonstrates that YW3-56 induces substantial metabolic reprogramming—characterized by reversal of the Warburg effect—and promotes a differentiated phenotype in NB4 APL cells. This is accompanied by inhibition of AKT/mTOR signaling activity and activation of apoptotic pathways. Our findings suggest that the multi-modal anti-leukemic effects of YW3-56 may arise from its dual action: targeting PAD4-mediated epigenetic regulation and disrupting AKT-driven metabolic pathways, ultimately leading to enhanced differentiation and apoptosis. Therefore, the PAD4-AKT signaling axis represents a promising candidate for further investigation as a potential target in combination therapeutic strategies for APL.

## 4. Materials and Methods

### 4.1. Chemicals

The PAD4 inhibitor YW3-56 was synthesized via a conventional liquid-phase method reported previously. The solid powder was dissolved in dimethyl sulfoxide (DMSO; Sigma-Aldrich, San Diego, CA, USA) and diluted with suitable media for further use in experiments. The anti-PAD4 (ab96758), anti-H3Cit (R2+R8+R17) (ab5103), Alexa Fluor^®^ 568 goat anti-rabbit IgG H&L (ab175471), and Alexa Fluor^®^ 488 anti-GLUT1 (ab195359) antibodies were obtained from Abcam Biotechnology (Cambridge, UK). The PE anti-human CD11b Antibody (#393111) and APC anti-human CD14 Antibody (#399205) were purchased from Biolegend (San Diego, CA, USA). The anti-PI3K (#4292), anti-phospho-PI3 kinase (Ser249; #13857), anti-AKT (#9271), anti-phospho-AKT (Thr308; #9275), anti-mTOR (#2972), and anti-phospho -mTOR (Ser2448; #2971) antibodies were obtained from Cell Signaling (Danvers, MA, USA). 2-NBD-glucose (2-NBDG), the calcein-AM/PI cell viability assay kit, and the Annexin V-FITC apoptosis detection kit were purchased from Beyotime Institute (Nanjing, Jiangsu, China).

### 4.2. PAD4 Inhibition

The PAD4-inhibitory activity of YW3-56 was evaluated using a colorimetric assay based on PAD4-catalyzed citrullination of *N*-α-benzoyl-*L*-arginine ethyl ester (BAEE). Various concentrations of YW3-56 were added to 96-well plates with 10× buffer; then, the PAD4 enzyme was added, and the mixture was incubated at 37 °C for 60 min. BAEE was then added, and the mixture was incubated at 37 °C for 90 min. The reaction was quenched with 5 mM HClO_4_, and 125 μL of the mixture was subsequently coincubated with Reagents A and B at 100 °C for 30 min. The absorbance at 465 nm was measured, and the IC_50_ values were calculated using GraphPad Prism 9.5.1.

### 4.3. Cell Culture

The human promyelocytic leukemia cell line NB4 and HL-60 were acquired from the Institute of Biochemistry and Cell Biology, Chinese Academy of Sciences (Shanghai, China). NB4 cells were cultured in RPMI-1640 medium (HyClone), and HL-60 cells were cultured in IMDM medium (Gibco, Grand Island, NY, USA), each supplemented with 10% fetal bovine serum (FBS; Gibco) and 1% penicillin/streptomycin solution (Gibco). All cells were maintained in an incubator with 5% CO_2_ atmosphere at 37 °C.

### 4.4. Cell Viability Assay

The cytotoxicity of YW3-56, AIRA, or their combination to NB4 cells was evaluated via an MTT assay. Briefly, NB4 cells were seeded at a density of 5 × 10^3^ cells per well in 96-well plates and incubated overnight. The cells were then treated with PBS (as a control) or various concentrations of YW3-56 for 48 h, with medium containing an equivalent concentration of DMSO serving as a control. The cells were incubated for an additional 4 h after the MTT reagent was added. The absorbance was then measured at 490 nm using a full-wavelength microplate reader (Thermo Scientific™ Multiskan GO, Waltham, MA, USA).

### 4.5. Live/Dead Cell Assay

NB4 cells were seeded in six-well plates at a density of 1 × 10^5^ cells per well and incubated for 4 h. The cells were then treated with PBS or various concentrations of YW3-56 (8, 4, and 2 μM) for 24 h. Following the Calcein-AM/PI assay kit instructions, a staining mixture containing Calcein-AM and PI was prepared. The cells were incubated with the staining mixture for 30 min at 37 °C. The viable cells were stained green by Calcein-AM, while the dead cells were stained red by propidium iodide (PI). Cell viability was assessed using a FACS Calibur flow cytometer (BD Bioscience, Franklin Lakes, NJ, USA).

### 4.6. Cell Apoptosis Assay

Apoptosis was assessed using an Annexin V-FITC apoptosis detection kit. NB4 cells were seeded in six-well plates at a density of 1 × 10^5^ cells per well and incubated for 4 h. The cells were treated with PBS or various concentrations of YW3-56 for 24 h. After two washes with PBS, the cells were resuspended in binding buffer and incubated with 5 μL of annexin V-FITC and 10 μL of PI in the dark for 15 min at room temperature. Following double staining, a total of 10,000 cells per sample were analyzed using a FACS Calibur flow cytometer.

### 4.7. Cellular H3Cit Immunofluorescence Assay

NB4 cells were seeded in confocal dishes at a density of 1 × 10^5^ cells per well and allowed to adhere overnight. After treatment with PBS or 4 μM YW3-56 for 48 h, the cells were washed with PBS and fixed in 4% paraformaldehyde containing 0.2% Triton X-100 for 15 min at 4 °C. Following blocking with 5% BSA for 1 h at room temperature, the cells were incubated overnight at 4 °C with anti-H3Cit (R2+R8+R17) antibody (1: 400), followed by Alexa Fluor^®^ 568 goat anti-rabbit IgG H&L (1:800) for 1 h at room temperature in the dark. Nuclear DNA was counterstained with Hoechst 33342 for 3 min. Fluorescence images were captured using a laser confocal microscope (TCS SP8 STED, Leica, Wetzlar, Germany), and mean fluorescence intensity was quantified via ImageJ 1.46a.

### 4.8. Western Blot Analysis

Western blotting was performed to analyze the expression of PAD4 protein and the PI3K/AKT/mTOR pathway in NB4 cells. Cells treated with PBS or various concentrations of YW3-56 were collected, and lysed in ice-cold RIPA lysis buffer with a 1% phosphatase and protease inhibitor mixture. Protein concentrations were quantified using the BCA assay. The proteins were then mixed with loading buffer and heated at 100 °C for 10 min. A total of 20 μg of each protein sample was separated by 12% SDS–PAGE and transferred onto PVDF membranes (Merck Millipore, Darmstadt, Germany). The membranes were blocked with 5% BSA for 2 h at room temperature, followed by three washes with Tris-buffered saline containing 0.1% Tween-20 (TBST) and incubation with the target primary antibodies overnight at 4 °C. After the primary antibodies were removed, the membranes were incubated with the following horseradish peroxidase-conjugated secondary antibodies: goat anti-rabbit IgG (ZB-2301; Zsbio, Beijing, China) and goat anti-mouse IgG (ZB-2305; Zsbio, Beijing, China) in the dark for 1 h at room temperature. The protein signal was visualized and captured using an enhanced chemiluminescence system (Thermo Fisher Scientific, OL191210A, Waltham, MA, USA). Protein expression levels were quantified via ImageJ 1.46a.

### 4.9. Mass Cytometry

After incubation with different concentrations of YW3-56 for 24 h, the NB4 cells were collected and cultured with Cell-IDTM cisplatin (Fluidigm, South San Francisco, CA, USA) for 2 min to distinguish living cells. FIX I solution (Fluidigm) was then used to fix the cells for 15 min. After three washes with Cell Staining Buffer (CSB, Fluidigm), the cells were stained with a surface marker antibody cocktail for 30 min at room temperature. Following another three washes with CSB, the cells were permeabilized with Perm-S (Fluidigm), washed three times with CSB, and stained with an intracellular marker antibody cocktail.

MaxPar^®^ Barcoding (Fluidigm) was used to conjugate the purified antibodies (as listed in the Supplemental Information). All the metal-conjugated antibodies were titrated to the optimal concentration before staining the cells. After three washes with CSB, the cells were stained with an iridium-containing DNA intercalator (191 Ir/193 Ir, 125 nM final concentration) in Fix and Perm solution (Fluidigm) for 1 h at room temperature. The cells were then washed three times with double-distilled water and resuspended in a 10% sEQ Four Element Calibration Beads (Fluidigm) before detection with a HeliosTM mass cytometer (Fluidigm).

All normalized flow cytometry standard (.fcs) files were uploaded to Cytobank (https://www.cytobank.org/) for data cleaning and analysis.

### 4.10. Maturation-Related Cell Surface Differentiation Antigen Expression Assay 

NB4 cells were treated with 4 μM YW3-56, 10 nM ATRA, or their combination for 5 days. After treatment, cells were harvested by centrifugation at 500× *g* for 5 min, fol-lowed by two washes with pre-chilled PBS. The cells were then resuspended in PBS containing 2% FBS and incubated with PE anti-human CD11b Antibody (3:50 dilution) and APC anti-human CD14 Antibody (3:50 dilution) for 20 min at 37 °C in the dark. After staining, cells were washed once with PBS, resuspended in 300 μL PBS, and immediately analyzed on a FACS Calibur flow cytometer.

### 4.11. RNA and Protein Preparation

Briefly, NB4 cells were incubated with 4 μM YW3-56 in 10 cm dishes for 24 h. The cells were scraped off with a cell scraper and collected by centrifugation at 500× *g* for 5 min. One milliliter of TRIzol reagent (Magen, Guangzhou, China) was added to 1.5 mL EP tubes to obtain total RNA, and 1 mL of RIPA lysis buffer was added to obtain total protein. RNA sequencing experiments were performed by Novegene Company (Beijing, China), while proteomics data were obtained at the Basic Research Institute of Peking Union Medical College Hospital. The detailed methods are provided in the Supplementary Files.

### 4.12. GLUT1 Expression Assay

After being seeded in six-well plates at a density of 1 × 10^5^ cells per well and incubated for 4 h, the cells were treated with various concentrations of YW3-56 for 24 h. Following two washes with PBS, the cells were fixed with 80% methanol for 5 min and permeabilized with 0.1% PBS-Tween for 20 min. The cells were incubated with 5% BSA blocking solution for 1 h, followed by incubation with an antibody (ab195359, 1:500 dilution) for 30 min at room temperature. The nuclear DNA was stained with PI for 3 min, after which the cells were washed with PBS. Finally, cell staining was detected using a high-content image flow cytometry (Amnis^®^ ImageStream Mk II, Luminex, Austin, TX, USA) and statistically analyzed by IDEAS 6.2. The intrinsic fluorescence of YW3-56, imaged using the same channel as DAPI, was used to monitor its cellular localization.

### 4.13. 2-NBDG Glucose Uptake Assay

NB4 cells were seeded in confocal dishes at a density of 1 × 10^5^ cells per well in RPMI 1640 medium without FBS. The cells were treated with various concentrations of YW3-56 for 2 h. Then, PBS containing 2-NBDG was added to each group at a final concentration of 50 μM, and the mixture was incubated for 30 min at 37 °C. After two washes with PBS to remove excess 2-NBDG, the cells were imaged with a laser confocal microscope, and the mean fluorescence intensity was statistically analyzed via ImageJ 1.46a.

### 4.14. Statistical Analyses

The collected data were analyzed using GraphPad Prism 9.5.1 and are presented as the mean ± SD. Statistical significance was determined via one-way ANOVA Tukey’s test or Student’s two-tailed *t*-test. Significance levels are indicated as follows: *, *p* < 0.05; **, *p* < 0.01; ***, *p* < 0.001. All results were confirmed in a minimum of three independent experiments.

## Figures and Tables

**Table 1:** Assessment of the quality of the transcriptomic data from NB4 cells.

Sample	Raw Reads	Clean Reads	Error Rate (%)	Q20 (%)	Q30 (%)	GC Content (%)
control_1	40,956,314	39,955,562	0.03	97.27	92.33	53.04
control_2	47,137,690	45,968,034	0.03	97.37	92.53	52.69
control_3	46,282,384	44,900,684	0.03	97.35	92.46	52.34
YW3-56_1	45,422,734	44,242,556	0.03	97.44	92.66	51.56
YW3-56_2	46,369,202	45,278,048	0.03	97.46	92.74	52.08
YW3-56_3	45,600,868	44,619,054	0.03	97.23	92.27	51.49

Notes: Q20 (%) and Q30 (%) represent the percentage of bases with Phred values greater than 20 and 30, respectively, among the total bases. GC content (%): represents the percentage of G and C bases among all bases.

## Data Availability

The datasets of RNA Seq presented in this study are openly available in NCBI at http://www.ncbi.nlm.nih.gov/bioproject/764844 (accessed on 20 January 2025), reference number PRJNA764844. The proteomics data presented in this study are openly available in iProX at http://proteomecentral.proteomexchange.org (accessed on 20 January 2025), reference number PXD028892.

## Supplementary Materials

The following supporting information can be downloaded at: https://www.mdpi.com/article/10.3390/ph18111646/s1, Supplementary Methods; Supplementary Data: Figure S1. Structure identification of YW3-56. (A) Chemical structure and 1HNMR, (B) HR-MS, (C) HPLC spectrum of YW3-56; Figure S2. The PAD4 inhibitor YW3-56 suppresses (A) PAD4 enzymatic activity and (B) NB4 cell viability; Figure S3. Single-cell mass cytometry analysis of apoptosis markers in YW3-56-treated NB4 cells; Figure S4. The PAD4 inhibitor YW3-56 inhibits cell proliferation and induces apoptosis in HL-60 cells. (A) Effect of YW3-56 on HL-60 cell viability assessed by MTT assay. (B–C) Representative results and quantitative analysis of YW3-56-induced apoptosis in HL-60 cells detected by Annexin V-FITC/PI staining. Data are presented as mean + SD. ***, *p* < 0.001; Table S1. Panel of single-cell mass cytometry; Figure S5. Therapeutic and differentiation potential of YW3-56 co-administered with ATRA in NB4 cells. (A) Effect of YW3-56 combined with ATRA on NB4 cell viability assessed by MTT assay. (B–C) Flow cytometric analysis of cell-surface differentiation antigen CD11b/CD14 expression in NB4 cells following 5-day treatment with 4 μM YW3-56, 10 nM ATRA, or their combination. Data are presented as mean ± SD. ***, *p* < 0.001; Figure S6. The top 25 GO annotated entries of the co-expressed DEGs/DEPs from YW3-56-treated NB4 cells; Figure S7. GO enrichment bubble diagram of the co-expressed DEGs/DEPs from YW3-56-treated NB4 cells in the three GO branches. up-up, co-upregulated DEGs/DEPs. down-down, co-downregulated DEGs/DEPs. The top 25 significantly enriched pathways are plotted, with the ordinate representing pathway categories and the abscissa indicating the enrichment ratio (differentially expressed molecules/total annotated entries). Bubble size corresponds to differential molecule count, while color intensity reflects statistical significance (deeper red = lower *p*-values); Figure S8. KEGG enrichment bubble diagram of the co-expressed DEGs/DEPs from YW3-56-treated NB4 cells. up-up, co-upregulated DEGs/DEPs. down-down, co-downregulated DEGs/DEPs. gene-down-protein-up, down-regulated DEGs and up-regulated DEPs; Figure S9. (A) Proteomic quantification showed upregulation of PIK3AP1 in YW3-56-treated NB4 cells; (B) Mass cytometry analysis demonstrated reduced AKT phosphorylation.

## Abbreviations

The following abbreviations are used in this manuscript:

2-NBDG	2-NBD-Glucose
6PGD	6-phosphogluconate dehydrogenase
AKT	Alpha serine/threonine-protein kinase
APL	Acute promyelocytic leukemia
ATRA	All-trans-retinoic acid
ATO	Arsenic trioxide
DEGs	differentially expressed genes
DEPs	differentially expressed proteins
DMSO	Dimethyl sulfoxide
Eno1	Enolase 1
G6PD	Glucose-6-phosphate 1-dehydrogenase
GLUT1	Glucose transporter 1
H3Cit	Histone 3 citrullination
IDH3B	Isocitrate dehydrogenase [NAD] subunit beta
IDH3G	Isocitrate dehydrogenase [NAD] subunit gamma
LDHA	Lactate Dehydrogenase A
LSCs	Leukemia stem cells
mTOR	Mechanistic target of rapamycin
NET	Neutrophil extracellular trap
PAD4	Protein arginine deiminase 4
PCA	Principal component analysis
PEPCK	Phosphoenolpyruvate carboxykinase
PI3K	Phosphatidylinositol 3-kinase
PKM2	Pyruvate kinase M2
TCA	Tricarboxylic acid
