# Supplementary material for: Reversing the Warburg Effect: YW3-56 Induces Leukemia Differentiation via AKT-Mediated Glucose Metabolic Reprogramming
Source: Pharmaceuticals (Basel). 2025 Oct 31;18(11):1646. doi: 10.3390/ph18111646 (PMC12655338; doi:10.3390/ph18111646)
Supplement: Supplementary file 1 [file pharmaceuticals-18-01646-s001.zip › pharmaceuticals-3793148-supplementary.pdf]

Supplementary Information for

Reversing the Warburg Effect: YW3-56 Induces Leukemia Differentiation via AKT-Mediated Glucose Metabolic Reprogramming

Di Zhu [1], Dan Gao [1], Yu Lu [2,3], Na Chen [1], Li Zhang [1], Lan Zhang [2,*] and Yuji Wang [2,3,*]

1 Department of Pharmacy, Xuanwu Hospital, Capital Medical University, Beijing 100053, China

2 Department of Medicinal Chemistry, College of Pharmaceutical Sciences, Capital Medical University, Beijing 100069, China

3 Beijing Key Laboratory of Drug Innovation for Neuro-Oncology, Beijing Engineering Research Center of Targeted Drugs and Cell Therapy for CNS Tumors, Beijing 100069, China

* Correspondence: wangyuji@ccmu.edu.cn (Yuji Wang), xwzhanglan@126.com (Lan Zhang)

---

1. Supplementary Methods

1.1 RNA Sequencing methods

RNA quantification and qualification

RNA integrity was assessed using the RNA Nano 6000 Assay Kit of the Bioanalyzer 2100 system (Agilent Technologies, CA, USA).

Library preparation for Transcriptome sequencing

A total amount of 1 µg RNA per sample was used as input material for the RNA sample preparations. Briefly, mRNA was purified from total RNA using poly-T oligo-attached magnetic beads. Fragmentation was carried out using divalent cations under elevated temperature in First Strand Synthesis Reaction Buffer (5X). First strand cDNA was synthesized using random hexamer primer and M-MuLV Reverse Transcriptase (RNase H-). Second strand cDNA synthesis was subsequently performed using DNA Polymerase I and RNase H. Remaining overhangs were converted into blunt ends via exonuclease/polymerase activities. After adenylation of 3’ ends of DNA fragments, Adaptor with hairpin loop structure were ligated to prepare for hybridization. In order to select cDNA fragments of preferentially 370~420 bp in length, the library fragments were purified with AMPure XP system (Beckman Coulter, Beverly, USA). Then PCR was performed with Phusion High-Fidelity DNA polymerase, Universal PCR primers and Index (X) Primer. At last, PCR products were purified (AMPure XP system) and library quality was assessed on the Agilent Bioanalyzer 2100 system.

Clustering and sequencing (Novogene Experimental Department)

The clustering of the index-coded samples was performed on a cBot Cluster Generation System using TruSeq PE Cluster Kit v3-cBot-HS (Illumina) according to the manufacturer's instructions. After cluster generation, the library preparations were sequenced on an Illumina Novaseq platform and 150 bp paired-end reads were generated.

Quality control

Raw data (raw reads) of fastq format were firstly processed through in-house perl scripts. In 1 this step, clean data (clean reads) were obtained by removing reads containing adapter, reads containing poly-N and low quality reads from raw data. At the same time, Q20, Q30 and GC content the clean data were calculated. All the downstream analyses were based on the clean data with high quality.

Reads mapping to the reference genome

Reference genome and gene model annotation files were downloaded from genome website directly. Index of the reference genome was built using Hisat2 v2.0.5 and paired-end clean reads were aligned to the reference genome using Hisat2 v2.0.5. We selected Hisat2 as the mapping tool for that Hisat2 can generate a database of splice junctions based on the gene model annotation file and thus a better mapping result than other non-splice mapping tools.

Quantification of gene expression level

featureCounts v1.5.0-p3 was used to count the reads numbers mapped to each gene. And then FPKM of each gene was calculated based on the length of the gene and reads count mapped to this gene. FPKM, expected number of Fragments Per Kilobase of transcript sequence per Millions base pairs sequenced, considers the effect of sequencing depth and gene length for the reads count at the same time, and is currently the most commonly used method for estimating gene expression levels.

Differential expression analysis

(For DESeq2 with biological replicates) Differential expression analysis of two conditions/groups (two biological replicates per condition) was performed using the DESeq2 R package (1.20.0). DESeq2 provide statistical routines for determining differential expression in digital gene expression data using a model based on the negative binomial distribution. The resulting P-values were adjusted using the Benjamini and Hochberg's approach for controlling the false discovery rate. Genes with an adjusted P-value <0.05 found by DESeq2 were assigned as differentially expressed.

(For edgeR without biological replicates) Prior to differential gene expression analysis, for each sequenced library, the read counts were adjusted by edgeR program package through one scaling normalized factor. Differential expression analysis of two conditions was

performed using the edgeR R package (3.22.5). The P values were adjusted using the Benjamini & Hochberg method. Corrected P-value of 0.05 and absolute foldchange of 1.5 were set as the threshold for significantly differential expression.

Enrichment analysis of differentially expressed genes

Gene Ontology (GO) enrichment analysis of differentially expressed genes was implemented by the clusterProfiler R package, in which gene length bias was corrected. GO terms with corrected Pvalue less than 0.05 were considered significantly enriched by differential expressed genes. KEGG is a database resource for understanding high-level functions and utilities of the biological system, such as the cell, the organism and the ecosystem, from molecular-level information, especially large-scale molecular datasets generated by genome sequencing and other high-through put experimental technologies (http://www.genome.jp/kegg/). We used clusterProfiler R package to test the statistical enrichment of differential expression genes in KEGG pathways. The Reactome database brings together the various reactions and biological pathways of human model species. Reactome pathways with corrected Pvalue less than 0.05 were considered significantly enriched by differential expressed genes. The DO (Disease Ontology) database describes the function of human genes and diseases. DO pathways with corrected Pvalue less than 0.05 were considered significantly enriched by differential expressed genes. The DisGeNET database integrates human disease-related genes. DisGeNET pathways with corrected Pvalue less than 0.05 were considered significantly enriched by differential expressed genes. We used clusterProfiler software to test the statistical enrichment of differentially expressed genes in the Reactome pathway, the DO pathway, and the DisGeNET pathway.

Gene Set Enrichment Analysis

Gene Set Enrichment Analysis (GSEA) is a computational approach to determine if a pre-defined Gene Set can show a significant consistent difference between two biological states. The genes were ranked according to the degree of differential expression in the two samples, and then the predefined Gene Set were tested to see if they were enriched at the top or bottom of the list. Gene set enrichment analysis can include subtle expression changes. We use the local version of the GSEA analysis tool http://www.broadinstitute.org/gsea/index.jsp, GO, KEGG, Reactome, DO and DisGeNET 3 data sets were used for GSEA independently.

SNP analysis

GATK2 (v3.7) software was used to perform SNP calling. Raw vcf files were filtered with GATK standard filter method and other parameters (cluster:3; WindowSize:35; QD < 2.0 o; FS > 30.0; DP < 10.

AS analysis

Alternative Splicing is an important mechanism for regulate the expression of genes and the variable of protein. rMATS(3.2.5) software was used to analysis the AS event.

PPI analysis of differentially expressed genes

PPI analysis of differentially expressed genes was based on the STRING database, which known and predicted Protein-Protein Interactions.

Fusion Analysis

Fusion gene refers to the chimeric gene formed by the fusion of all or part of the sequences of two genes, which is generally caused by chromosome translocation, deletion and other reasons. We used Starfusion software (1.2.0) to detect genes that are fused. Star-fusion is a software package uses fusion output results of STAR alignment to detect fusion transcripts, including SATR alignment, SATRfusion. predict, SATR-fusion.filter was used to correct the predicted results of Star-fusion to ensure the accuracy of the results.

Weighted correlation network analysis

WGCNA (Weighted correlation network analysis) is a systematic biological method used to describe the gene association modes among different samples. it can be used to identify gene sets that are highly synergistic changed, and identify candidate biomarkers or therapeutic targets based on the coherence of gene sets and the correlation between gene sets and phenotypes. The R package WGCNA is a set of functions used to calculate various weighted association analysis, which can be used for network construction, gene screening, gene cluster identification, topological feature calculation, data simulation and visualization. WGCNA is suitable for multisample data. Generally, more than 15 samples are required. One input file is sample information, that is, a matrix describing the traits of the sample: the traits used for association analysis must be numeric; If it is a regional or categorical variable, it needs to be 4 converted to a 0-1 matrix. The other is gene expression data. For transcriptome sequencing, FPKM can be used as gene expression data.

1.2 Proteomics methods

Protein digest

Proteins were reduced with 10 mM dithiothreitol and alkylated with 55 mM iodoacetamide. Then, proteins were equivalented with 20 mM Tris.HCl buffer on 30 KD ultracentrifugation tube and digested with trypsin (modified sequencing grade; Promega, Madison, WI, USA) overnight at 37 °C in ultracentrifugation tube. The tryptic peptides were de-salted and concentrated on reverse phase C18 StageTips. The elution products dry down in a vacuum centrifuge to remove solvent.

LC-MS/MS

Peptides were revolved in 0.1% FA and separated with EASY-n LC1000 system. Column oven was set to 60 °C. Peptides were delivered to a trap column (75 µm × 2 cm, C18, 5µm, Thermo Scientific), then separated with capillary LC column (75 µm × 100 mm, C18, 3 µm, Kyoto Monotech). The eluted gradient was 6-28% for 48 min and 28-95% for 4 min buffer B (0.1% formic acid, 100% ACN; flow rate, 0.6 µL/min). An Orbitrap Fusion Lumos mass spectrometer was used to analyze the eluted peptides from LC. The data was acquired with data-independent acquisition acquisition under high-sensitivity mode using the following parameters: Positive mode was set. One cycle contains one full scan and 40 segments fragment scans. Full scan range is from 350 to 1300 m/z and screened at 120,000 resolution. Fragment spectra were collected at 3,000 resolution and segmented 19 MS/MS scans. Maximum injection time is 50msec.

Proteomics data analysis

Raw data of DIA were analyzed by Spectronaut (version 14.3, Biognosys) with default settings. The Spectral libraries of DIA was generated using total raw data files with Q value cutoff of 0.01 and minimum of six fragment ions. The raw files were searched against the Human database downloaded from reviewed Swissprot. Decoy items were generated by inverse mode. The samples were quantitative evaluation basing on the MS2 area. Cross run were normalized according to the global abundance area.

2. Supplementary Data

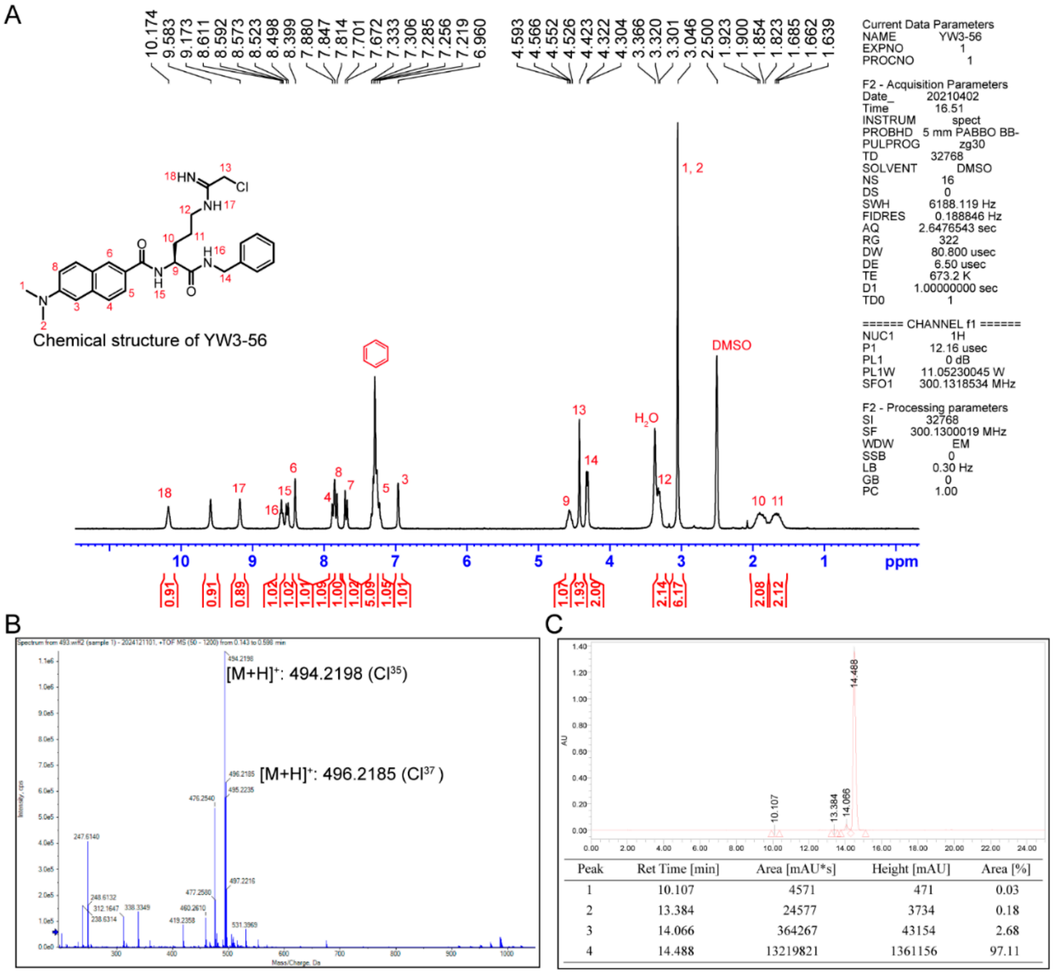

Peak	Ret Time [min]	Area [mAU*s]	Height [mAU]	Area [%]
1	10.107	4571	471	0.03
2	13.384	24577	3734	0.18
3	14.066	364267	43154	2.68
4	14.488	13219821	1361156	97.11

Figure S1. Structure identification of YW3-56. (**A**) Chemical structure and ^{1}HNMR, (**B**) HR-MS, (**C**) HPLC spectrum of YW3-56.

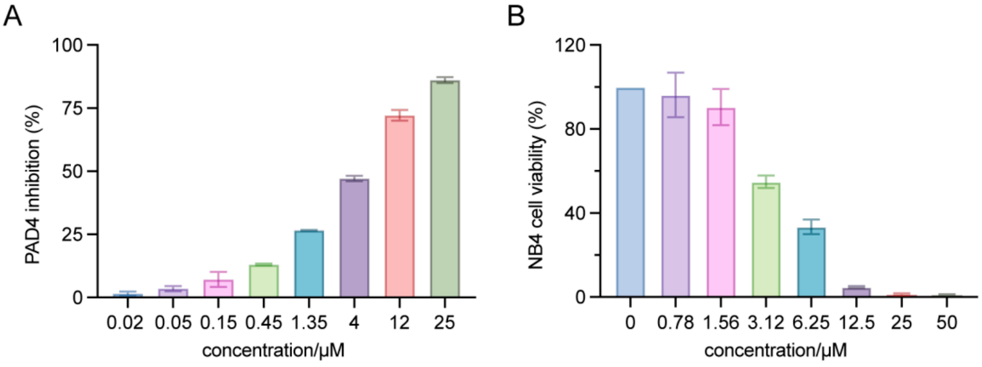

Figure S2. The PAD4 inhibitor YW3-56 suppresses (**A**) PAD4 enzymatic activity and (**B**) NB4 cell viability.

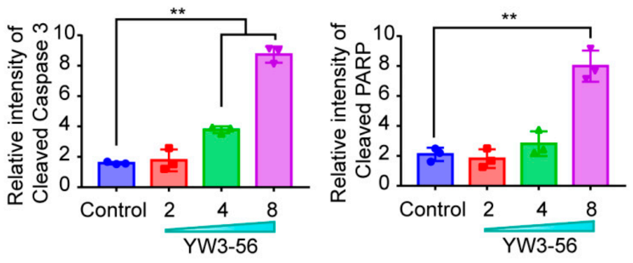

**, $p < 0.01$

Figure S3. Single-cell mass cytometry analysis of apoptosis markers in YW3-56-treated NB4 cells.

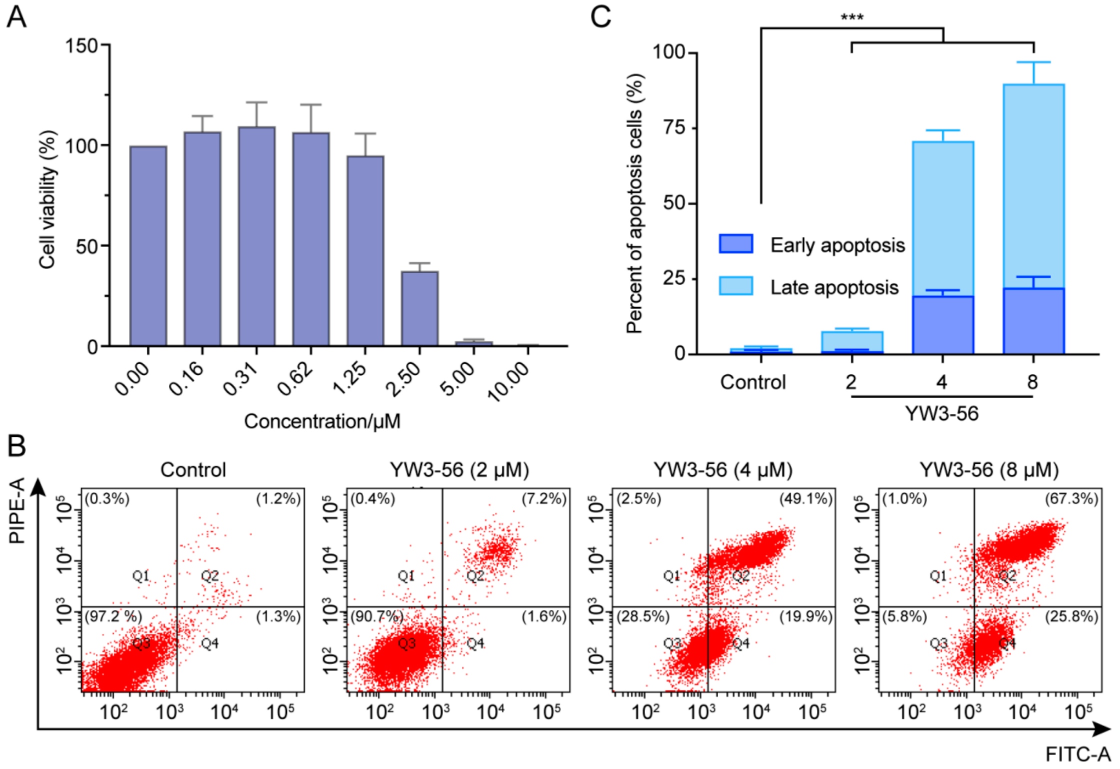

Figure S4. The PAD4 inhibitor YW3-56 inhibits cell proliferation and induces apoptosis in HL-60 cells. (**A**) Effect of YW3-56 on HL-60 cell viability assessed by MTT assay. (**B,C**) Representative results and quantitative analysis of YW3-56-induced apoptosis in HL-60 cells detected by Annexin V-FITC/PI staining. Data are presented as mean ± SD. ***, $p < 0.001$.

Table S1. Panel of single-cell mass cytometry

Antibodies	Metal	Clone	Company
nanog	142	16H3A48	biolegend
CLEAVED PARP	143	F21-852	FLUIDIGM
P53	144	BP53-12	Biolegend
CD133	145	170411	RD
IFNα/βRα	146	SC7391	SANTA CRUZ BIOTECHNOLOGY
CD39	147	498403	RD
CD14	148	RMO52	FLUIDIGM
SYK	149	4D10.2	FLUIDIGM
p-AKT	150	545007	abcam
C-MYC	151	9E/10	biolegend
AKT	152	D9E	biolegend
CD86	153	IT2.2	BIOLEGEND
PD-1	155	EH12.2H7	FLUIDIGM
P38	156	D3F9	FLUIDIGM
P21	159	12D1	FLUIDIGM
CYCLIN D1	160	SP4	abcam
HISTONE H3	161	11D8	abcam
PAD4	162	094H5	biolegend
TGF-beta	163	TW4-6H10	FLUIDIGM
CD44	164	BJ18	biolegend
PD-L1	165	EPR15759	abcam
ATG5	166	9474	SANTA CRUZ BIOTECHNOLOGY
EIF2S1	167	EPR23098-50	abcam
CD15	168	W6D3	BIOLEGEND
ATF4	169	739441	RD
CD49F	170	GOH3	RD
CD80	171	37711	RD
CLEAVED CASPASE3	172	5A1E	FLUIDIGM
p-P38	173		SANTA CRUZ BIOTECHNOLOGY
CD38	174	HB-7	BIOLEGEND
PS6	175	N7-548	sciences
p-P53	176	SC377567	SANTA CRUZ BIOTECHNOLOGY
CD11b	209	ICRF44	FLUIDIGM

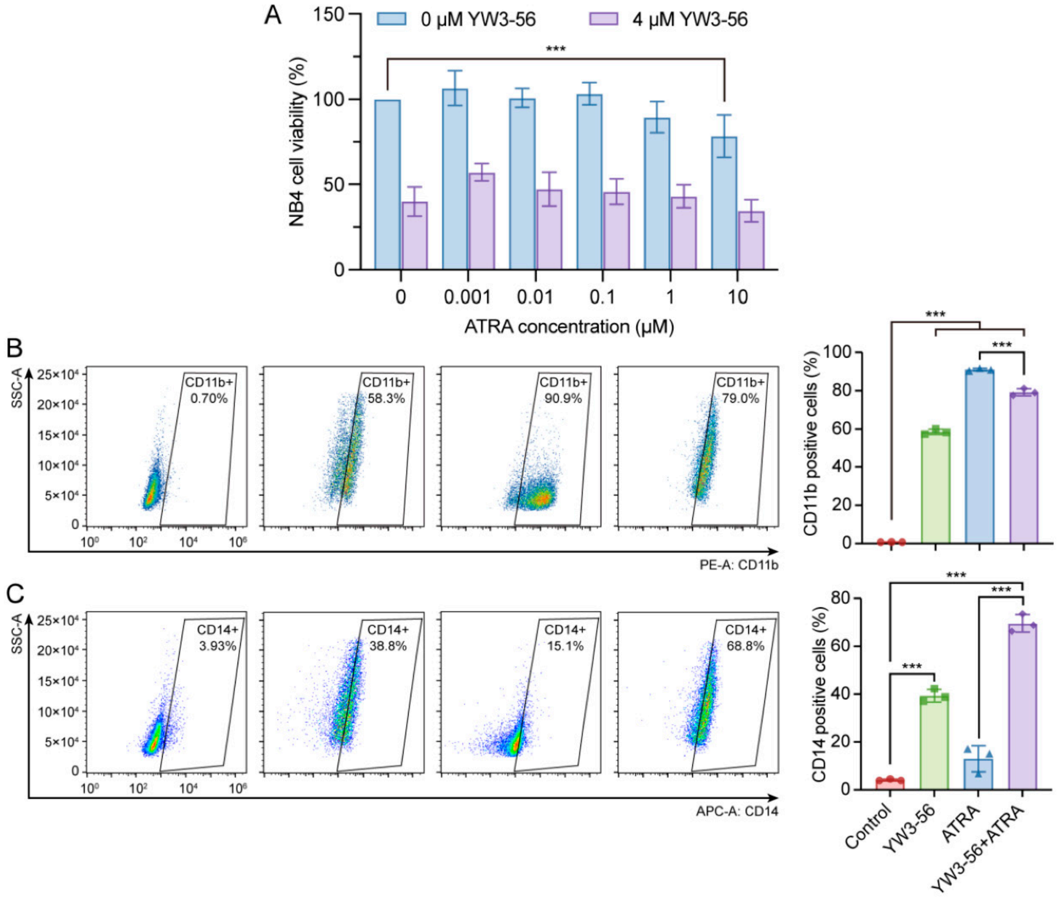

Figure S5. Therapeutic and differentiation potential of YW3-56 co-administered with ATRA in NB4 cells. (**A**) Effect of YW3-56 combined with ATRA on NB4 cell viability assessed by MTT assay. (**B**–**C**) Flow cytometric analysis of cell-surface differentiation antigen CD11b/CD14 expression in NB4 cells following 5-day treatment with 4 µM YW3-56, 10 nM ATRA, or their combination. Data are presented as mean ± SD. ***, $p < 0.001$.

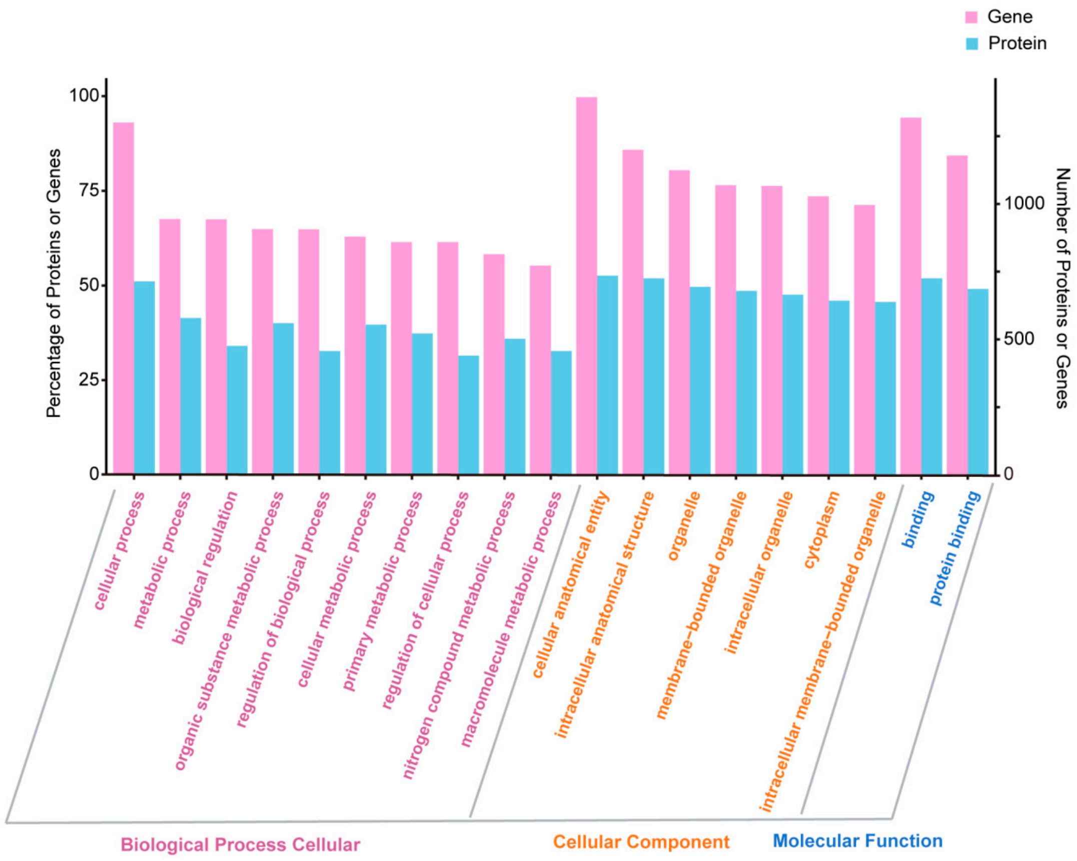

Figure S6. The top 25 GO annotated entries of the co-expressed DEGs/DEPs from YW3-56-treated NB4 cells.

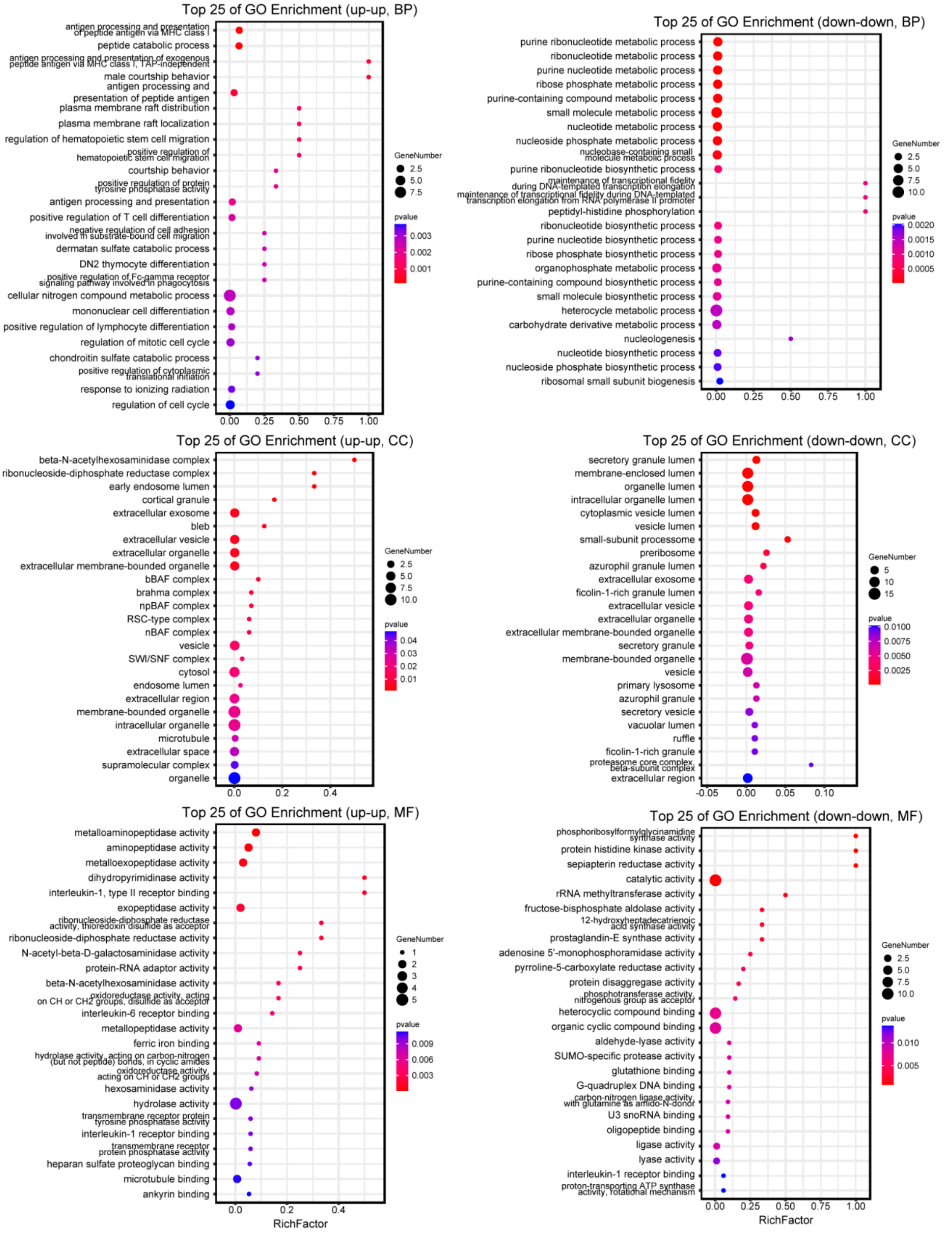

Figure S7. GO enrichment bubble diagram of the co-expressed DEGs/DEPs from YW3-56-treated NB4 cells in the three GO branches. up-up, co-upregulated DEGs/DEPs. down-down, co-downregulated DEGs/DEPs. The top 25 significantly enriched pathways are plotted, with the ordinate representing pathway categories and the abscissa indicating the enrichment ratio (differentially expressed molecules/total annotated entries). Bubble size corresponds to differential molecule count, while color intensity reflects statistical significance (deeper red = lower *p*-values).

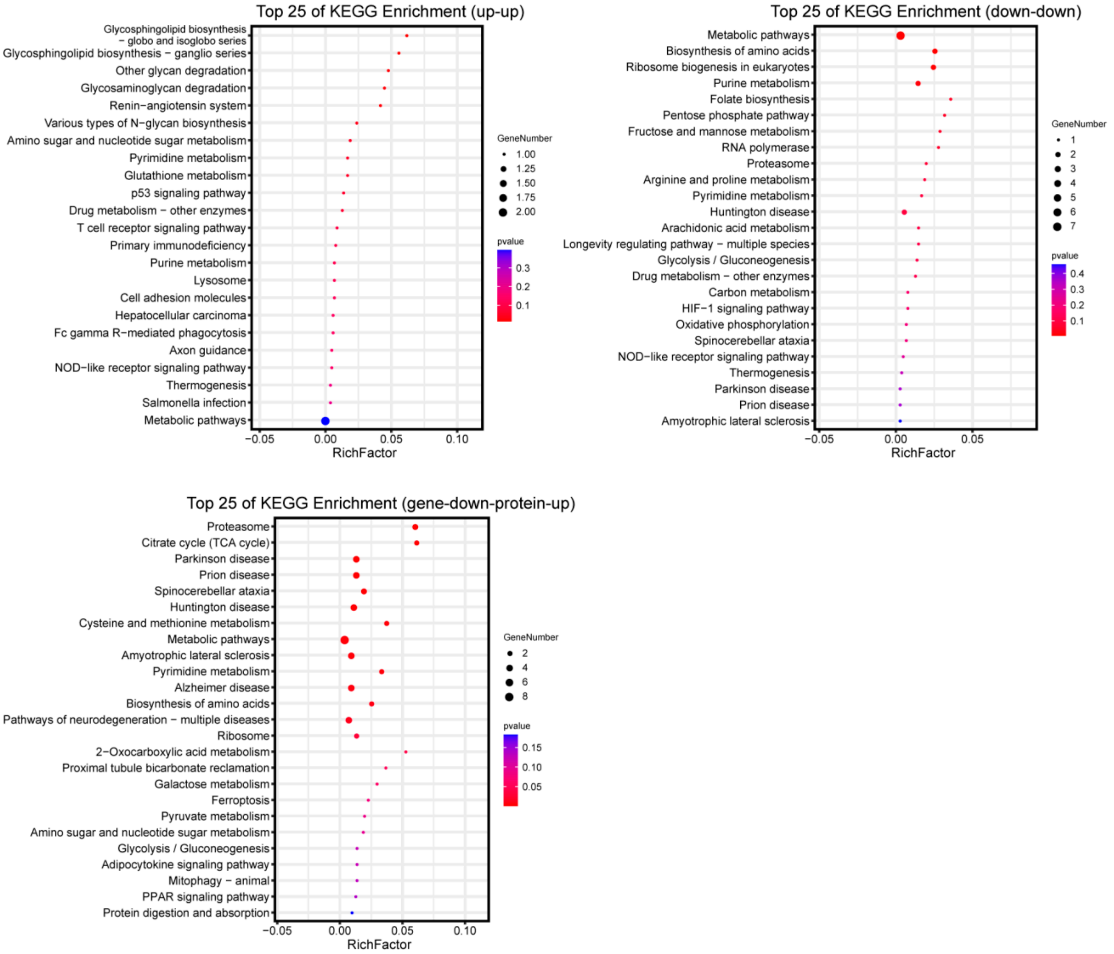

Figure S8. KEGG enrichment bubble diagram of the co-expressed DEGs/DEPs from YW3-56-treated NB4 cells. up-up, co-upregulated DEGs/DEPs. down-down, co-downregulated DEGs/DEPs. gene-down-protein-up, down-regulated DEGs and up-regulated DEPs.

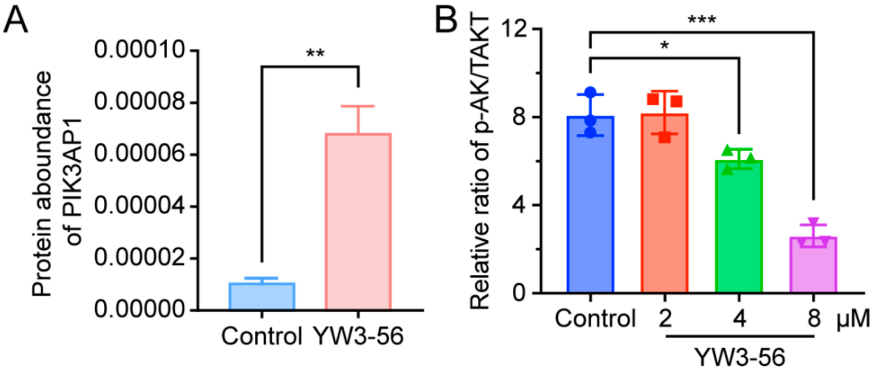

*, $p < 0.05$; ***, $p < 0.01$.

Figure S9. (**A**) Proteomic quantification showed upregulation of PIK3AP1 in YW3-56-treated NB4 cells; (**B**) Mass cytometry analysis demonstrated reduced AKT phosphorylation.
